# Clinical outcomes and safety of tocilizumab in severe fever with thrombocytopenia syndrome: a retrospective multicenter cohort study

**DOI:** 10.3389/fcimb.2026.1894849

**Published:** 2026-09-09

**Authors:** Quanman Hu, Yehan Jin, Yaming Wei, Jundong Chen, Yan Hu, Yanyan Yang, Li Zhang, Shuaiyin Chen, Guangcai Duan

**Affiliations:** 1 College of Public Health, Zhengzhou University, Zhengzhou, China; 2 Huaiyang Disease Control and Prevention Center, Zhoukou, China; 3 Disease Control and Prevention Center, Xinyang, China

**Keywords:** outcome, PSM, retrospective, safety, SFTS, tocilizumab

## Abstract

**Background:**

Severe fever with thrombocytopenia syndrome (SFTS) is an emerging tick - borne zoonotic infectious disease. Severe SFTS cases exhibit an extremely high fatality rate, but there remain no effective clinical treatments.

**Methods:**

Propensity Score Matching (PSM) and logistic regression analysis were used to assess the efficacy, safety, and baseline factors influencing patient prognosis of tocilizumab in the treatment of SFTS.

**Results:**

A total of 833 SFTS cases were included, of whom 171 received tocilizumab. After PSM, a higher recovery rate was observed in the tocilizumab group than in the control group (76.61% vs 67.70%, *P* = 0.039). However, the between-group difference in in-hospital mortality did not reach statistical significance after matching (16.96% vs 15.22%, *P* = 0.322). Regarding selected adverse events (AEs) such as co-infection, the between-group differences did not reach statistical significance; these represent observed differences in retrospectively recorded data and should not be interpreted as evidence of safety. Multivariable logistic regression showed that age ≥ 65 years, vasopressor therapy, platelet transfusion, and Ct value were associated with prognosis among tocilizumab-treated patients, with vasopressor use and platelet transfusion likely reflecting disease severity rather than independent causal determinants.

**Conclusion:**

In this retrospective cohort, after PSM, tocilizumab use was associated with a higher recovery rate in SFTS patients, although the between-group difference in in-hospital mortality did not reach statistical significance. Observed differences in selected AEs were based on retrospectively recorded data and should not be interpreted as evidence of safety. Given the exploratory nature, prospective studies are warranted.

## 1 Introduction

Severe fever with thrombocytopenia syndrome (SFTS) is an emerging tick - borne zoonotic infectious disease, mainly caused by Dabie bandavirus (also known as SFTSV), which belongs to the Phenuiviridae family within the order Bunyavirales (Sun et al., 2025). This disease was initially discovered in China in 2009 and subsequently became prevalent in other Asian regions such as South Korea and Japan (Yu et al., 2011; Takahashi et al., 2014; Yun et al., 2014). The clinical manifestations of SFTS include acute fever, accompanied by thrombocytopenia and leukopenia, and can result in various clinical outcomes. While most patients exhibit mild symptoms, some cases may progress to severe illness or even death. Reportedly, the fatality rate ranges from 14.6% to 17.8%, with an average fatality rate of 16.2% (Li et al., 2018). Particularly in severe cases of SFTS, such as those complicated by encephalitis, the fatality rate can reach as high as 44.7% (Cui et al., 2015). Currently, there is no effective therapy available for SFTS.

Currently, numerous drugs have been reported for use in the treatment of SFTS. A single-blind, randomized controlled trial on the antiviral drug favipiravir (T-705) for SFTS therapy indicated that oral administration of Favipiravir in combination with supportive therapy could effectively lower the mortality rate. Nevertheless, the observed reduction in the high viral load group did not reach statistical significance when compared with the control group (Li et al., 2021). Additionally, glucocorticoids (GC) have been systematically recommended as an adjuvant therapy for patients with severe SFTS in the guidelines issued by the National Health Commission of China (Chen et al., 2022). However, a retrospective cohort study implies that GC therapy might actually elevate the mortality rate of patients with severe SFTS, and the immunosuppression it induces may also heighten the risk of triggering infections (Chen et al., 2024). Meanwhile, the Chinese expert consensus also recommends intravenous immunoglobulin (IVIG) as an adjuvant therapy for severe SFTS. The primary reason could be that IVIG is mainly employed to supplement antibodies or regulate the immune response in inflammatory or autoimmune diseases. At present, the conclusions of different studies are inconsistent, and its efficacy remains debatable (Liu et al., 2023; Zhang et al., 2023). Finally, other therapies such as therapeutic plasma exchange (TPE) have demonstrated certain potential by rapidly eliminating viruses and inflammatory factors from the bloodstream (Zhang et al., 2021).

Clinicians consider cytokine storm as one of the crucial aspects in the pathogenic process of severe SFTS. Hence, blocking pro - inflammatory cytokines could be a promising therapeutic approach, which may facilitate the recovery from multiple organ function damage by mitigating immune responses. A recent clinical trial has reported that tocilizumab (an IL - 6 antagonist) can lower the mortality rate of patients with severe SFTS. Results from dynamic IL - 6 level monitoring have indicated that the use of tocilizumab is significantly negatively correlated with the rapid decline of IL – 6 (Ge et al., 2024). Some small - sample observational studies have also demonstrated that tocilizumab therapy yields better outcomes than TPE (Yoo et al., 2025). Nevertheless, in actual clinical practice, the individual conditions of SFTS patients vary, and they cannot be strictly enrolled and evaluated as in clinical trials. Therefore, this study assessed the efficacy, safety, and baseline factors influencing patient prognosis of tocilizumab in the treatment of SFTS in the real world via a multicenter, observational, retrospective cohort study, offering a basis for clinical guidelines.

## 2 Methods

### 2.1 Study design and participants

This study systematically collected the clinical data of patients diagnosed with SFTS in multiple hospitals in Xinyang City, China, from January 2023 to December 2024. According to Chinese law and the “Diagnosis and Treatment of Fever with Thrombocytopenia Syndrome (2024 Edition)”, SFTS cases must be reported to the China Information System for Disease Control and Prevention (CISDCP) within 24 hours after diagnosis, and polymerase chain reaction (PCR) verification should be carried out by the Xinyang Center for Disease Control and Prevention to rule out false positives. In addition, the Xinyang Center for Disease Control and Prevention will also conduct retrospective medical record collection and follow - up on these confirmed SFTS cases and then organize the data into a database. This study strictly adheres to the ethical principles stated in the Helsinki Declaration. The research protocol was reviewed and approved by the Ethics Committee of Zhengzhou University (Approval Number: ZZUIRB2024 - 205). As a retrospective study, all data have been anonymized, and no collection of personal identity information was involved. Therefore, the ethics committee waived the requirement of informed consent.

The diagnostic criteria for confirming SFTS must satisfy any one of the following conditions: 1, Positive SFTSV nucleic acid; 2, Isolation and culture of SFTSV from clinical specimens; 3, A four - fold or greater increase in the titer of SFTSV - IgG from the acute phase to the convalescent phase or during seroconversion. In addition, tocilizumab therapy entails collecting clear therapy instructions regarding the disease course in the case information system.

### 2.2 Data collection

This study collected demographic data, comorbidities, treatment methods of patients with SFTS. Laboratory results and the initial Ct value (viral load), days from symptom onset to hospitalization, and initial clinical classification were also collected. It is important to note that all variables used for PSM had complete data with no missing values. Missingness was confined to certain continuous laboratory parameters used for univariable and multivariable logistic regression. These missing laboratory data were handled using multiple imputation by chained equations (MICE) under the missing-at-random assumption, with predictive mean matching for continuous variables. We generated 20 imputed datasets and combined estimates using Rubin’s rules. Both treatment assignment (tocilizumab use) and the primary outcome (recovery/poor outcome) were included in the imputation model to minimize bias.

Regarding tocilizumab administration, because of the retrospective design, tocilizumab use was based entirely on the treating physician’s discretion and not on a predefined protocol. All treated patients received tocilizumab as a single intravenous infusion of 400 mg over 1 hour. A subset of patients received a second 400-mg dose 8–24 hours later if the attending physician judged the clinical response as insufficient. In clinical practice, tocilizumab was typically initiated when patients were progressing from mild/moderate to severe illness or had persistent severe disease.

### 2.3 Outcomes

In this study, the main clinical outcomes of SFTS patients after inpatient treatment were classified into two categories: Recovery and poor outcome. Among them, the criterion for recovery was determined by clinicians as the patient being basically cured of the disease, mainly based on the following: 1, The body temperature of SFTS patients returned to normal (axillary temperature ≤37.0 °C); 2, The platelet count returned to normal or above; 3, The patient reported no obvious abnormal conditions in the body, such as fatigue or pain.

The poor outcome was defined as any of the following: 1, in-hospital death from any cause; 2, transfer to another acute-care facility due to clinical deterioration; 3, discharge against medical advice with documented severe or moribund condition; 4, persistent severe SFTS at discharge (e.g., ongoing organ dysfunction, hemorrhage, or altered mental status that precluded de-escalation of care). Moreover, in all binary logistic regression analyses, poor outcome was modeled as the event (coded as 1, with favorable outcome/recovery coded as 0). Accordingly, an odds ratio (OR) below 1 indicates lower odds of poor outcome, which corresponds to a higher recovery rate and a more favorable clinical outcome. The secondary outcomes mainly included three types, namely disturbance of consciousness (apathy), mechanical ventilation, and length of stay (LOS) during hospitalization. Outcome adjudication was independently carried out by two investigators who were unaware of the treatment allocation. Discrepancies were resolved by a third reviewer. Data were retrieved from the electronic medical record using a pre - specified case - report form that concealed the treatment assignment.

### 2.4 Adverse events

AEs occurring after tocilizumab administration were identified from medical records and recorded as documented events without standardized severity grading; the main types included concurrent infections, respiratory symptoms, and digestive system symptoms. Additionally, the clinical outcomes of recovered SFTS patients at the time of discharge were analyzed.

### 2.5 Statistical analysis

#### 2.5.1 Propensity score matching

Given the non-randomized, retrospective design of this study, baseline characteristics differed between the tocilizumab and non-tocilizumab groups. To reduce potential confounding and improve covariate balance, we performed 1:2 nearest-neighbor PSM (tocilizumab: non-tocilizumab) without replacement, using a caliper of 0.1 on the logit of the propensity score. The propensity score was estimated using a logistic regression model (glm, family = binomial) that included the following baseline covariates: sex, age, viral load, days from symptom onset to hospitalization, comorbidities (hypertension, diabetes, cardiovascular disease, chronic liver disease, cancer), other diseases (excluding the above-listed comorbidities and acute illnesses), pre-tocilizumab treatments (favipiravir, ribavirin, antibiotics, platelet transfusion), and clinical classification. Standardized differences (SMD) were calculated to assess covariate balance after matching, with an absolute standardized difference <0.1 indicating acceptable balance. Subsequent comparative analyses of clinical outcomes were performed primarily in the matched cohort; unmatched results are also presented for transparency but should be interpreted with caution.

#### 2.5.2 Other statistical analyses

Statistical analyses were performed using R (v4.3.2). For the unmatched cohort, continuous variables were compared using independent-samples t-test (normally distributed) or Mann-Whitney U test (non-normal), and categorical variables were compared using χ² test or Fisher’s exact test (for expected frequencies <5). For the matched cohort (1:2 matching), all between-group comparisons accounted for the matched structure. For binary outcomes (e.g., recovery, mortality, AEs), conditional logistic regression was used, with each matched set treated as a separate stratum, to estimate OR and 95% confidence intervals (CIs) and to calculate P values.

Univariable and multivariable logistic regression analyses were conducted to identify baseline factors associated with prognosis. Variables with *P* < 0.05 in univariable analysis were entered into the multivariable model. All tests were two-sided, with statistical significance defined as *P* < 0.05.

## 3 Results

### 3.1 Patient inclusion process and basic characteristics of before and after PSM

**Figure 1:**
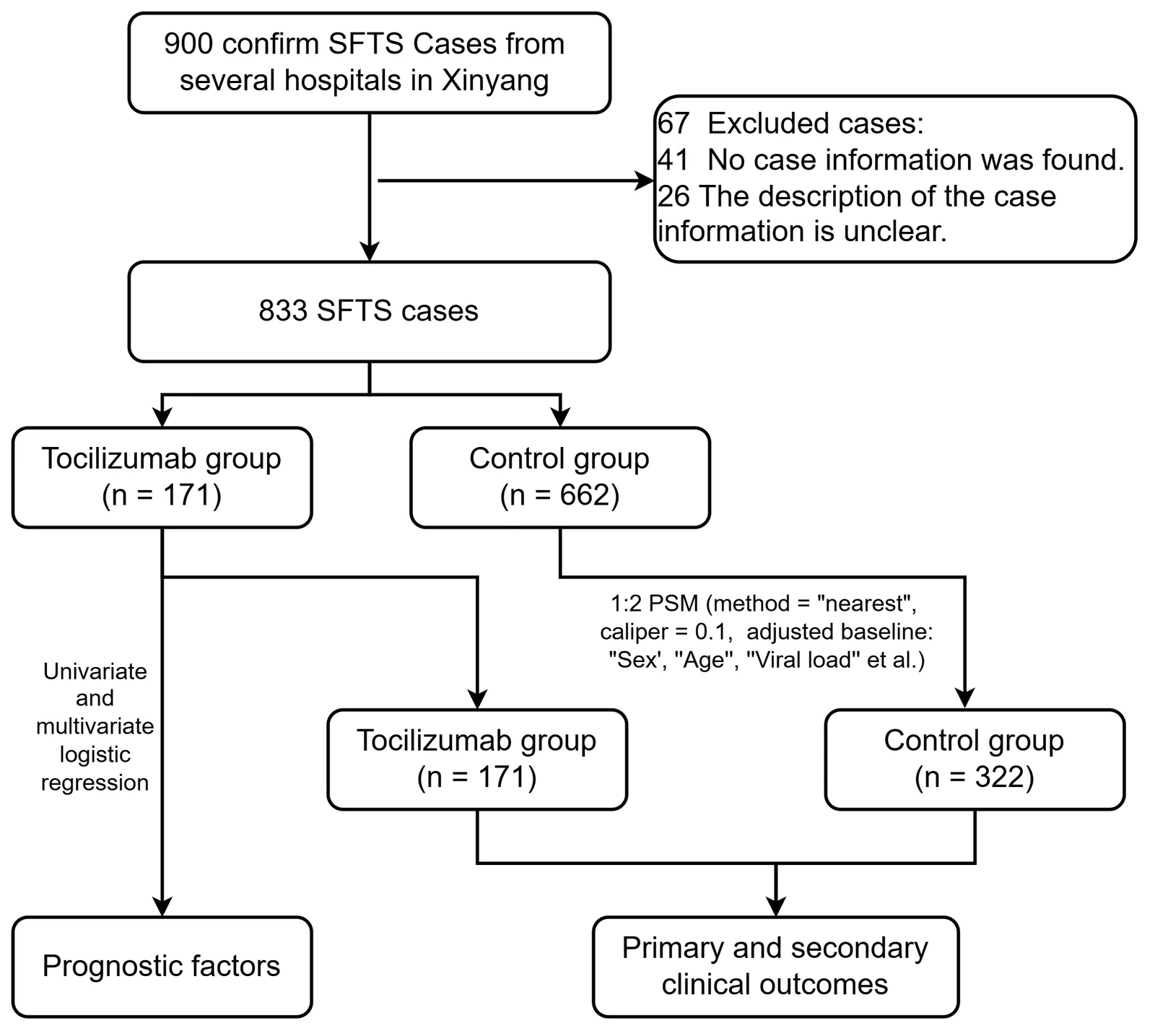
Flowchart of the screening process for eligible SFTS patients. Propensity score matching PSM.

A total of 900 patients diagnosed with SFTS were enrolled from January 2023 to December 2024 in this study. After initial screening, patients without medical record information (n = 41) or with ambiguous medical record descriptions (n = 26) were excluded. Consequently, a total of 833 SFTS patients were included in this study. Among them, 171 SFTS patients received tocilizumab therapy, whereas 662 SFTS patients did not. After PSM, 171 SFTS cases in the tocilizumab therapy group and 322 cases in the control group were included (**Figure 1**). In the tocilizumab treatment group, 17 patients (9.94%) received a second dose. The median timing of tocilizumab initiation relative to symptom onset and hospital admission was 7 (5 - 8) days and 2 (1 - 2) days, respectively.

**Figure 2:**
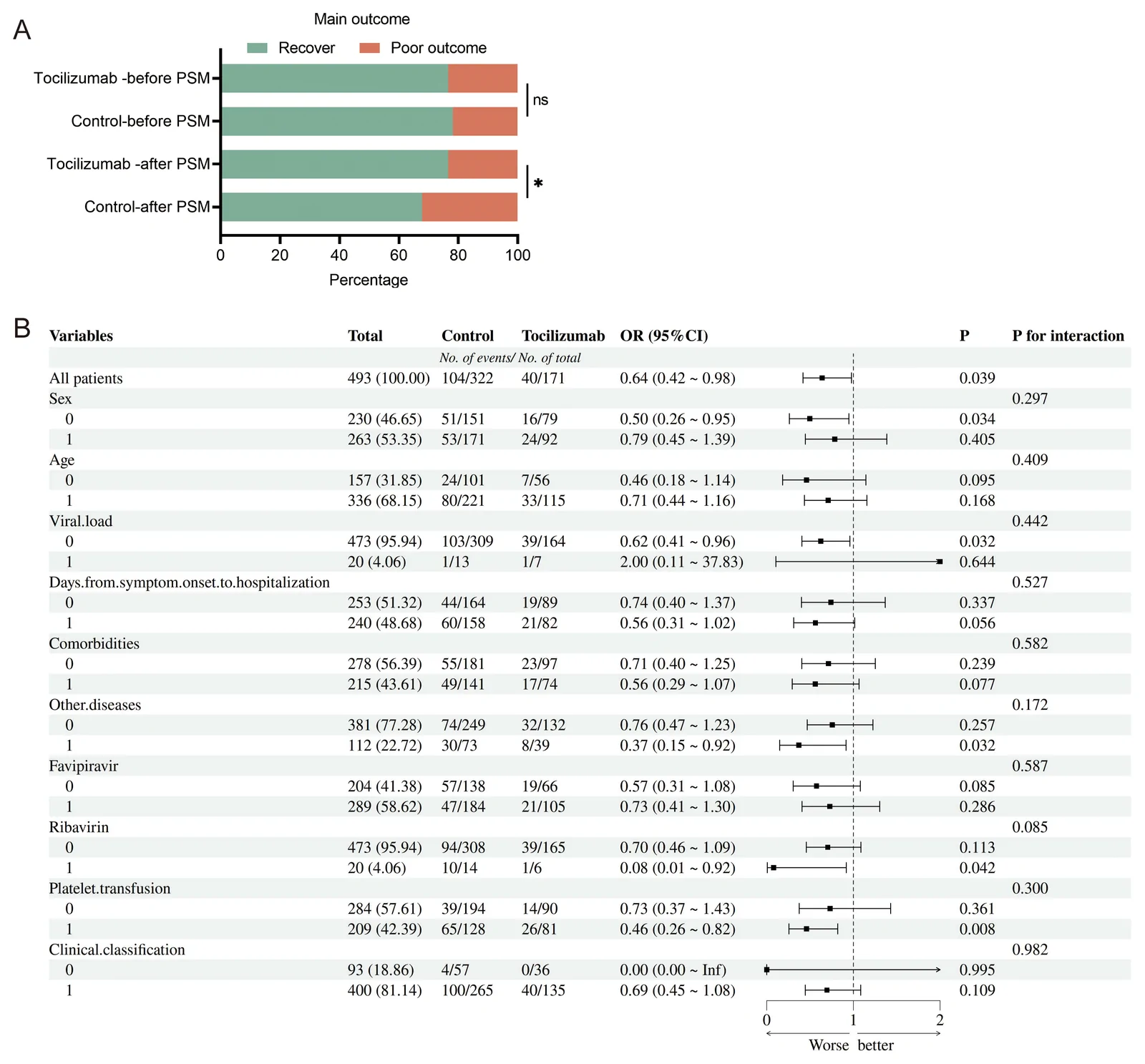
The primary outcome of tocilizumab therapy before and after PSM. **(A)** main outcome; **(B)** subgroup based on baseline characteristic of main outcomes: Gender (0: male, 1: female), age (0: < 65, 1: ≥65), viral load (0: low, 1: high), days from symptoms onset to hospitalization (0: short, 1: long), relative therapy (0: no, 1: yes), clinical classification (0: mild, 1: severe); ns not statistically significant, *P < 0.05.

As shown in **Table 1**, among the 833 included SFTS patients, the proportion of females was relatively high (59.18%), and the proportion of those aged 65 or above was also relatively high (63.99%). Subsequently, we compared the baseline information of the tocilizumab group and the control group before PSM and found that there were significant differences in many factors between the two groups. For example, there were differences in viral load (*P* < 0.001), days from symptom onset to hospitalization (*P* = 0.039), antibiotic therapy (*P* < 0.001), platelet transfusion (*P* < 0.001), etc. After PSM, the between-group differences in baseline characteristics did not reach statistical significance (*P* > 0.05). Meanwhile, **Supplementary Table 1** presented the SMD values of these factors before and after PSM. It was found that the differences in baseline factors were effectively controlled after PSM (SMD < 0.1), and the overall SMD value also decreased from 0.909 to 0.013. The love plot (**Supplementary Figure 1**) also confirmed this. Further subgroup analysis provided exploratory estimates for various predefined subgroups (**Figure 2B**), including sex (male: OR = 0.50, 95% CI: 0.26–0.95), viral load (low: OR = 0.62, 95% CI: 0.41–0.96), and underlying diseases (other diseases: OR = 0.37, 95% CI: 0.15–0.92). Additionally, exploratory estimates for tocilizumab combined with other therapies were as follows: ribavirin (OR = 0.08, 95% CI: 0.01–0.92) and platelet transfusion (OR = 0.46, 95% CI: 0.26–0.82). However, none of the treatment-by-subgroup interaction tests reached statistical significance (all P for interaction > 0.05). Therefore, these subgroup findings should be interpreted cautiously as purely descriptive; they do not provide evidence that the treatment effect of tocilizumab differs across these patient subgroups or concomitant therapies. No particular subgroup should be identified as more or less likely to benefit based on these data.

**Table 1:** The baseline characteristics of control and tocilizumab group before and after PSM.

Variable	Classification	Total (n = 833)	Control (n = 662)	Tocilizumab (n = 171)	*P*	Control (n = 322)	Tocilizumab (n = 171)	*P*
Gender	Male	340 (40.82)	261 (39.43)	79 (46.20)	0.108	151 (46.89)	79 (46.20)	0.883
	Female	493 (59.18)	401 (60.57)	92 (53.80)		171 (53.11)	92 (53.80)	
Age	< 65	300 (36.01)	244 (36.86)	56 (32.75)	0.318	101 (31.37)	56 (32.75)	0.754
	≥ 65	533 (63.99)	418 (63.14)	115 (67.25)		221 (68.63)	115 (67.25)	
Viral load	Low	717 (86.07)	553 (83.53)	164 (95.91)	<0.001	309 (95.96)	164 (95.91)	0.976
	High	116 (13.93)	109 (16.47)	7 (4.09)		13 (4.04)	7 (4.09)	
Days from symptom onset to hospitalization	Short	375 (45.02)	286 (43.20)	89 (52.05)	0.038	164 (50.93)	89 (52.05)	0.814
	Long	458 (54.98)	376 (56.80)	82 (47.95)		158 (49.07)	82 (47.95)	
Comorbidities	No	513 (61.58)	416 (62.84)	97 (56.73)	0.143	181 (56.21)	97 (56.73)	0.913
	Yes	320 (38.42)	246 (37.16)	74 (43.27)		141 (43.79)	74 (43.27)	
Other diseases	No	649 (77.91)	517 (78.10)	132 (77.19)	0.800	249 (77.33)	132 (77.19)	0.973
	Yes	184 (22.09)	145 (21.90)	39 (22.81)		73 (22.67)	39 (22.81)	
Favipiravir therapy	No	338 (40.58)	277 (41.84)	61 (35.67)	0.143	138 (42.86)	61 (35.67)	0.122
	Yes	495 (59.42)	385 (58.16)	110 (64.33)		184 (57.14)	110 (64.33)	
Ribavirin therapy	No	787 (94.48)	622 (93.96)	165 (96.49)	0.196	308 (95.65)	165 (96.49)	0.653
	Yes	46 (5.52)	40 (6.04)	6 (3.51)		14 (4.35)	6 (3.51)	
Antibiotic therapy	No	360 (43.22)	311 (46.98)	49 (28.65)	<0.001	89 (27.64)	49 (28.65)	0.811
	Yes	473 (56.78)	351 (53.02)	122 (71.35)		233 (72.36)	122 (71.35)	
Platelet transfusion	No	602 (72.27)	512 (77.34)	90 (52.63)	<0.001	194 (60.25)	90 (52.63)	0.103
	Yes	231 (27.73)	150 (22.66)	81 (47.37)		128 (39.75)	81 (47.37)	
Clinical classification	Mild	231 (27.73)	195 (29.46)	36 (21.05)	0.029	57 (17.70)	36 (21.05)	0.365
	Severe	602 (72.27)	467 (70.54)	135 (78.95)		265 (82.30)	135 (78.95)	

Propensity Score Matching PSM. Viral load: Low: virus Ct > 35, High: virus Ct < = 35; Days from symptom onset to hospitalization: Short: < 4 days, Long: >= 4 days; Comorbidities: including hypertension, diabetes, cardiovascular diseases, chronic liver disease, and cancer; other diseases (excluding the above-listed comorbidities and acute illnesses); Clinical classification: Mild cases and Severe cases according to Diagnosis and Treatment of Fever with Thrombocytopenia Syndrome (2024 Edition); The treatment-related variables (Favipiravir therapy, Ribavirin therapy, Antibiotic therapy, and Platelet transfusion) were initiated before tocilizumab administration.

### 3.2 The primary outcome of tocilizumab therapy

In this study, we primarily categorized the clinical outcomes of SFTS patients into two groups: recovery and poor outcomes, as depicted in **Figure 2A**. Prior to PSM, the recovery rate of the control group was 78.10%. The recovery rate of the tocilizumab group was 76.61%, and the difference between the groups did not reach statistical significance (*P* = 0.676). After PSM, the recovery rate of the tocilizumab group was significantly higher than that of the control group (76.61% vs 67.70%, *P* = 0.039), with an OR of 0.64 and 95% confidence interval (CI) of 0.42 - 0.98.

### 3.3 The secondary outcome of tocilizumab therapy

**Figure 3:**
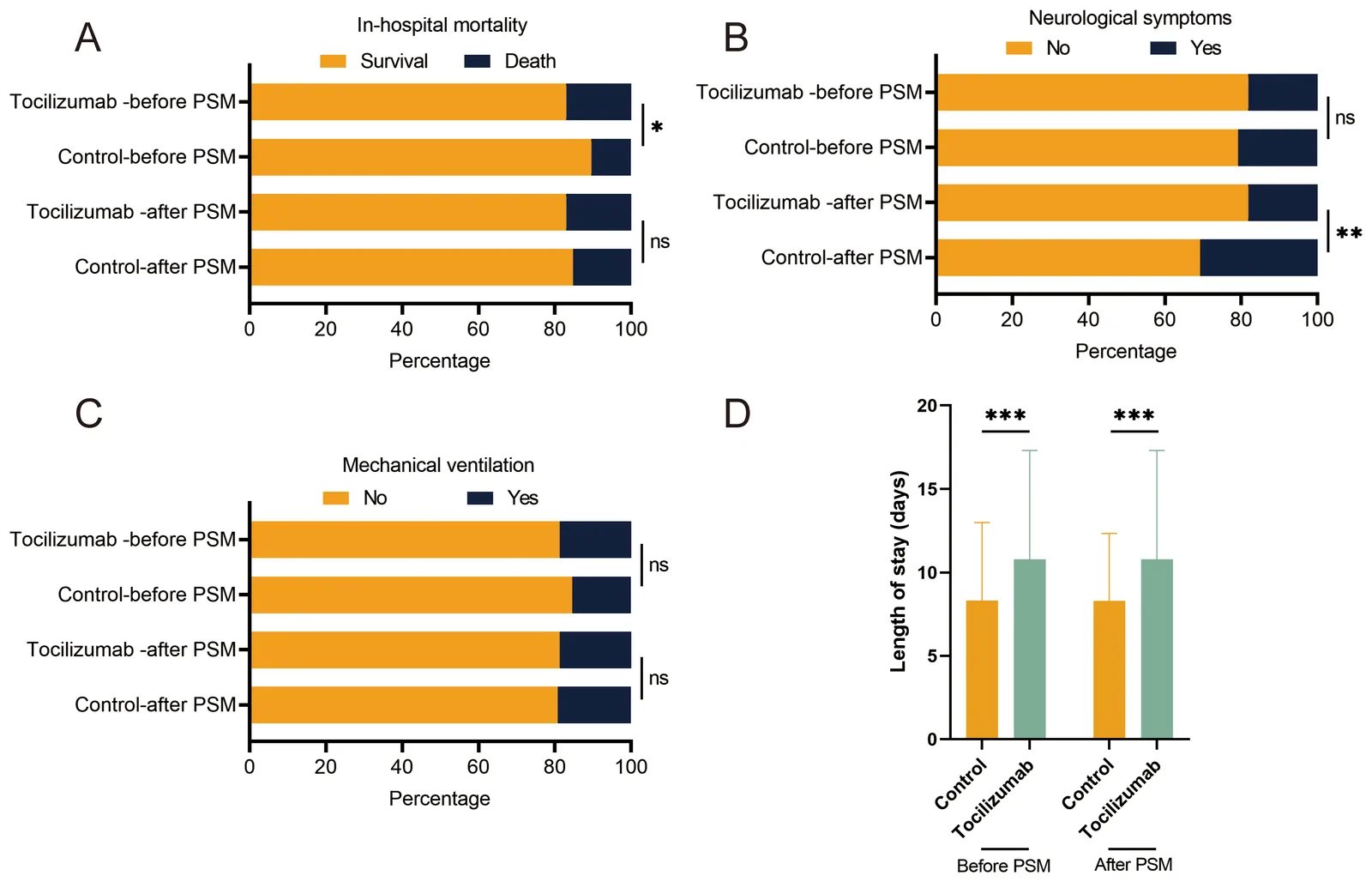
The secondary outcome of tocilizumab therapy before and after PSM. **(A)** In-hospital mortality; **(B)** neurological symptoms; **(C)**: mechanical ventilation; **(D)** Length of stay (days). ns not statistically significant, *P < 0.05, **P < 0.01, ***P < 0.001.

In addition, we also analyzed the differences in other clinical outcomes between the tocilizumab group and the control group before and after PSM. For example, regarding in-hospital mortality: before PSM, the in-hospital mortality rate in the tocilizumab group was significantly higher than that in the control group (16.96% vs 10.42%, *P* = 0.018). However, after PSM, the difference between the groups did not reach statistical significance (16.96% vs 15.22%, *P* = 0.322, **Figure 3A**). In addition, neurological symptoms including disturbance of consciousness or apathy: the control group showed a significant increase in the incidence of neurological symptoms after matching (*P* = 0.003, **Figure 3B**), but the between-group difference regarding mechanical ventilation did not reach statistical significance (*P* > 0.05, **Figure 3C**). Notably, regardless of whether before or after PSM, a longer LOS was observed in the tocilizumab group than in the control group (before PSM: 10.79 ± 6.52 vs 8.29 ± 4.05, *P* < 0.001; after PSM: 10.79 ± 6.85 vs 8.33 ± 4.66, *P* < 0.001, **Figure 3D**).

### 3.4 The safety analysis of tocilizumab therapy

**Figure 4:**
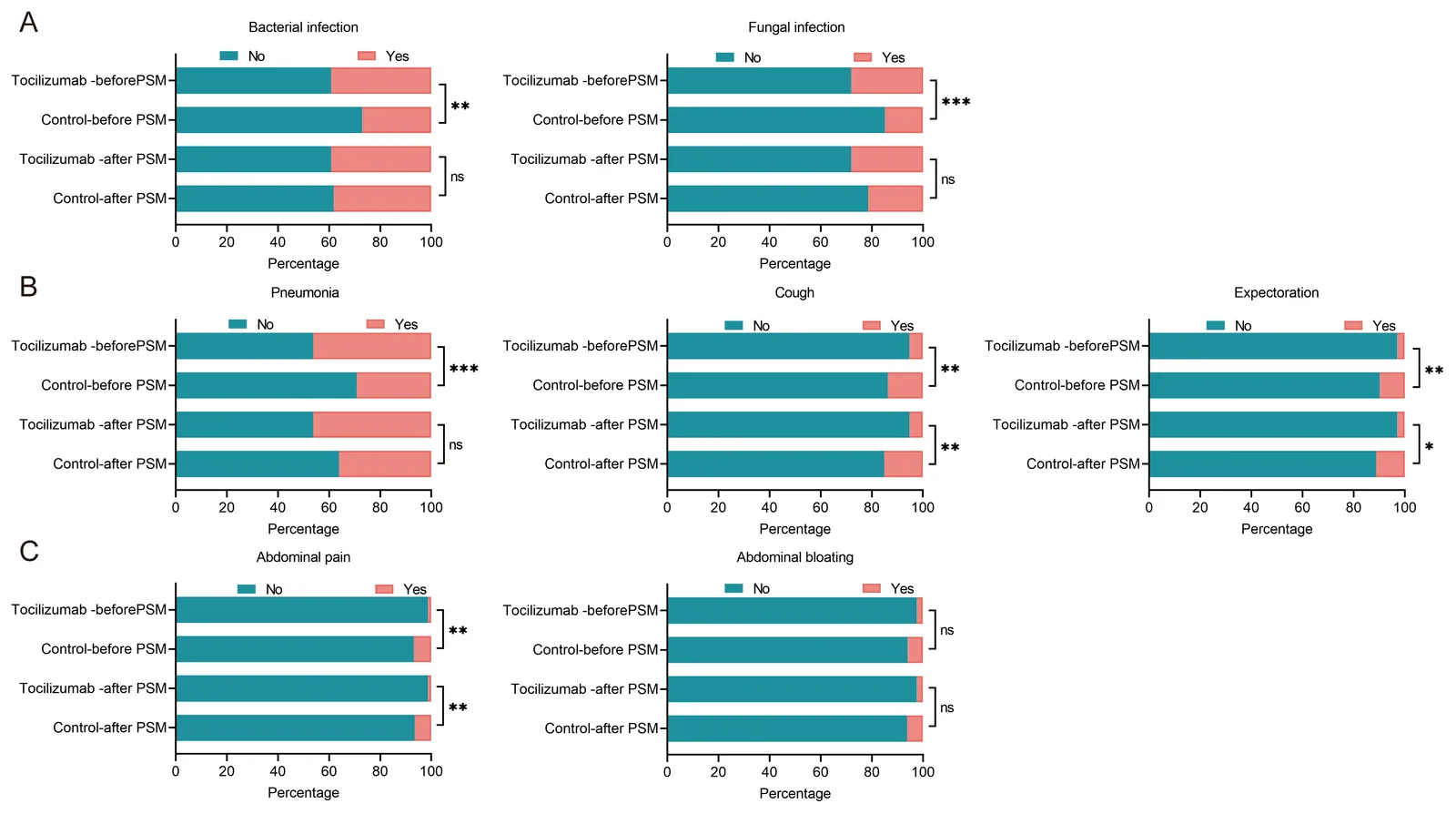
The safety analysis of tocilizumab therapy. **(A)** Co-infection including bacterial infection and fungal infection; **(B)** Respiratory system including pneumonia, cough and expectoration; **(C)** Digestive system including abdominal pain and abdominal bloating. ns not statistically significant, *P < 0.05, **P < 0.01, ***P < 0.001.

Next, we analyzed the potential AEs in the tocilizumab group. As depicted in **Figure 4A**, the incidence of concurrent infections in the tocilizumab group showed differences compared to the control group. Before PSM, the incidence rates of bacterial and fungal infections were higher in the tocilizumab group than in the control group (39.18% vs 27.04%, *P* = 0.002; 28.07% vs 14.80%, *P* < 0.001). However, after PSM, the between-group differences in the incidence rates of bacterial and fungal infections did not reach statistical significance (*P* = 0.831; *P* = 0.099). Regarding respiratory system, before PSM, the incidence rate of pneumonia was higher in the tocilizumab group than in the control group (*P* < 0.001), whereas the incidence rates of cough and expectoration were lower (*P* = 0.002; *P* = 0.004). After PSM, the between-group difference in the incidence rate of pneumonia did not reach statistical significance (*P* = 0.059), while the incidence rates of cough and expectoration remained lower than those in the control group (**Figure 4B**). Finally, for the digestive system, before PSM, the incidence rate of abdominal pain was lower than that in the control group (1.17% vs 6.80%, *P* = 0.004), while the between-group difference in the incidence rate of abdominal bloating did not reach statistical significance (5.89% vs 2.34%, *P* = 0.061, **Figure 4C**). After PSM, the incidence rates of abdominal pain in the tocilizumab group were lower than those in the control group (*P* = 0.007), while abdominal bloating did not reach statistical significance (*P* = 0.057, **Figure 4C**).

### 3.5 Prognostic factors of SFTS patients with tocilizumab therapy

Finally, we examined factors associated with prognosis (recovery and poor outcome) among SFTS patients treated with tocilizumab. A total of 40 of the 171 patients experienced a poor outcome. The univariable analysis (**Table 2**) showed that age ≥ 65 (OR = 2.82, 95% CI: 1.16–6.85, *P* = 0.022), vasopressor therapy (OR = 4.25, 95% CI: 1.73–10.45, *P* = 0.002), platelet transfusion (OR = 2.57, 95% CI: 1.23–5.36, *P* = 0.012), glucocorticoid/steroid therapy (OR = 2.33, 95% CI: 1.12–4.87, P = 0.024), and Ct value (OR = 0.85, 95% CI: 0.78–0.93, *P* < 0.001) were associated with prognosis. We then performed multivariable logistic regression including four predictors selected based on clinical importance and univariable analysis. The results showed that age ≥ 65 (OR = 3.30, 95% CI: 1.19–9.12, *P* = 0.021), vasopressor therapy (OR = 4.00, 95% CI: 1.48–10.75, *P* = 0.006), platelet transfusion (OR = 2.62, 95% CI: 1.16–5.95, *P* = 0.021), and Ct value (OR = 0.86, 95% CI: 0.79–0.95, P = 0.002) were associated with poor outcome among tocilizumab-treated patients. Of note, vasopressor therapy and platelet transfusion should be interpreted as markers of disease severity and clinical deterioration rather than as independent determinants of prognosis, and their associations may primarily reflect underlying critical illness.

**Table 2:** The factors associated with poor prognosis in patients with SFTS treated with tocilizumab.

Variables	Classification	Univariable analysis		Multivariable analysis	
		*P*	OR (95%CI)	*P*	OR (95%CI)
Gender	Female		1.00 (Reference)		
	Male	0.370	1.39 (0.68 ~ 2.85)		
Age	< 65		1.00 (Reference)		
	≥ 65	0.022	2.82 (1.16 ~ 6.85)	0.021	3.30 (1.19 ~ 9.12)
Clinical classification	Mild		1.00 (Reference)		
	Severe	0.987	0.00 (0.00 ~ Inf)		
Favipiravir therapy	No		1.00 (Reference)		
	Yes	0.188	0.62 (0.30 ~ 1.27)		
Ribavirin therapy	No		1.00 (Reference)		
	Yes	0.694	0.65 (0.07 ~ 5.70)		
Vasopressors	No		1.00 (Reference)		
	Yes	0.002	4.25 (1.73 ~ 10.45)	0.006	4.00 (1.48 ~ 10.75)
Platelet transfusion	No		1.00 (Reference)		
	Yes	0.012	2.57 (1.23 ~ 5.36)	0.021	2.62 (1.16 ~ 5.95)
GC/steroids therapy	No		1.00 (Reference)		
	Yes	0.024	2.33 (1.12 ~ 4.87)		
IVIG	No		1.00 (Reference)		
	Yes	0.059	0.38 (0.14 ~ 1.04)		
Antibiotic therapy	No		1.00 (Reference)		
	Yes	0.830	0.92 (0.42 ~ 2.00)		
Antifungal therapy	No		1.00 (Reference)		
	Yes	0.714	0.86 (0.39 ~ 1.90)		
Hypertension	No		1.00 (Reference)		
	Yes	0.829	0.91 (0.41 ~ 2.06)		
Diabetes	No		1.00 (Reference)		
	Yes	0.169	2.14 (0.72 ~ 6.30)		
Chronic liver disease	No		1.00 (Reference)		
	Yes	0.343	2.04 (0.47 ~ 8.95)		
Cardiovascular disease	No		1.00 (Reference)		
	Yes	0.177	0.35 (0.08 ~ 1.60)		
Cancer	No		1.00 (Reference)		
	Yes	0.685	1.65 (0.15 ~ 18.73)		
Other diseases	No		1.00 (Reference)		
	Yes	0.629	0.81 (0.34 ~ 1.93)		
CT		<.001	0.85 (0.78 ~ 0.93)	0.002	0.86 (0.79 ~ 0.95)
Days from symptom onset to hospitalization		0.154	0.90 (0.78 ~ 1.04)		
PT		0.564	1.02 (0.96 ~ 1.09)		
APTT		0.188	1.02 (0.99 ~ 1.06)		
FIB		0.629	1.00 (1.00 ~ 1.01)		
WBC		0.777	0.98 (0.83 ~ 1.15)		
NEUT%		0.223	1.02 (0.99 ~ 1.05)		
LYM%		0.366	0.98 (0.95 ~ 1.02)		
NEUT		0.838	1.02 (0.86 ~ 1.21)		
LYM		0.090	0.29 (0.07 ~ 1.22)		
RBC		0.642	0.96 (0.83 ~ 1.12)		
HGB		0.517	1.01 (0.99 ~ 1.03)		
PLT		0.510	1.00 (0.98 ~ 1.01)		
ALT		0.625	1.00 (0.99 ~ 1.00)		
GGT		0.703	1.00 (1.00 ~ 1.00)		
AST		0.784	1.00 (1.00 ~ 1.00)		
ALP		0.127	1.01 (1.00 ~ 1.01)		
TP		0.321	1.03 (0.97 ~ 1.09)		
ALB		0.619	1.02 (0.95 ~ 1.09)		
TPIL		0.236	1.05 (0.97 ~ 1.13)		
Cr		0.448	1.00 (1.00 ~ 1.01)		
TBA		0.163	1.02 (0.99 ~ 1.04)		
K		0.249	1.40 (0.79 ~ 2.47)		
Na		0.031	0.92 (0.85 ~ 0.99)		
LDH		0.354	1.00 (1.00 ~ 1.00)		
CK		0.173	1.00 (1.00 ~ 1.00)		

GC, glucocorticoids; IVIG, intravenous immunoglobulin; PT, prothrombin time; APTT, activated partial thromboplastin time; FIB, fibrinogen; WBC, white blood cell count; NEUT%, neutrophil percentage; LYM%, lymphocyte percentage; NEUT, neutrophil count; LYM, lymphocyte count; RBC, red blood cell count; HGB, hemoglobin; PLT, platelet count; ALT, alanine aminotransferase; GGT, gamma-glutamyl transferase; AST, aspartate aminotransferase; ALP, alkaline phosphatase; TP, total protein; ALB, albumin; TBIL, total bilirubin; Cr, creatinine; TBA, total bile acid; K, potassium; Na, sodium; LDH, lactate dehydrogenase; and CK, creatine kinase.

## 4 Discussion

Tocilizumab was initially approved for treating adult patients with moderate-to-severe active rheumatoid arthritis (RA) (Scott, 2017). It is a humanized monoclonal antibody that inhibits the binding of IL-6 by binding to both membrane - bound and soluble IL - 6 receptors. This action blocks IL - 6 signal transduction without interfering with the signal transduction of other IL - 6 family members (Mihara et al., 2005). Clinical research has reported that the initial IL - 6 levels in the non - surviving groups of SFTS cases in China and South Korea were significantly higher than those in the surviving groups (Yoo et al., 2021; Liu et al., 2025). Multivariable analysis demonstrated that the initial high IL - 6 level was associated with a poor prognosis in SFTS. IL - 6 has recently been recognized as a key cytokine in cytokine storms related to COVID - 19 and leukemia, particularly in cytokine storms induced by CAR T - cell therapy, where serum IL - 6 levels are extremely elevated (Grupp et al., 2013; Fajgenbaum and June, 2020). Meanwhile, cytokine storms have also been observed in SFTS deaths (Sun et al., 2012). A recent combined study of SFTS and COVID - 19 identified similar phenomena in severe COVID - 19 and SFTS patients: a large quantity of IL - 6 and IL - 10 was produced (Kang et al., 2023).

Moreover, compared with COVID - 19 patients treated with conventional therapy or placebo, tocilizumab therapy was associated with a lower 28 - day all - cause mortality rate (Shankar-Hari et al., 2021). However, the results of our study indicate that tocilizumab treatment did not significantly reduce the in-hospital mortality rate of SFTS patients. This might be because some patients who were discharged while still having severe SFTS died outside the hospital and were not included in the data. Notably, as IL-6 and other cytokine levels were not systematically measured in this study, we could not directly confirm the presence of a cytokine storm or adjust for them in our analysis; these mechanistic parallels should therefore be interpreted with caution. Finally, we found that tocilizumab therapy could significantly increase the LOS, which differs from the situation in COVID – 19 (Rezaei Tolzali et al., 2022). The possible reason is that this drug is less frequently used in SFTS cases, and clinicians extended the patients’ hospital stay to more comprehensively observe its efficacy and safety. Based on the above clinical outcomes and safety results of tocilizumab, which are consistent with the clinical study findings by Ge et al. and the observational results by Yoo et al., those previous studies reported a significant reduction in mortality with tocilizumab in severe SFTS (Ge et al., 2024; Yoo et al., 2025). However, in the present study, after PSM, the difference between the groups did not reach statistical significance. Next, we conducted an analysis of the AEs induced by tocilizumab therapy. The findings indicated that following PSM, the between-group differences did not reach statistical significance in selected AEs such as co-infection. Multiple studies on secondary infections in SFTS have revealed that steroid and TPE might lead to secondary bacterial infections (Ge et al., 2022; Seo et al., 2024; Hu et al., 2025). Regarding the respiratory system, the difference between the groups in the incidence of pneumonia did not reach statistical significance. Furthermore, the symptoms of cough and expectoration were alleviated. Some research proposes that pulmonary infections could be triggered by factors such as IVIG and autoimmune diseases (Zuo et al., 2023). The incidence of pneumonia is associated with the throat swab positivity rate of SFTSV (indicating severity) (Hu et al., 2024). Finally, regarding the digestive system, considering the low incidence in both groups, the results may be subject to bias, and large - sample studies are required in the future.

Finally, the multivariable analysis within the tocilizumab therapy group indicated that age ≥ 65, vasopressor therapy, platelet transfusion, and Ct value were associated with poor outcome among tocilizumab-treated patients. Previous studies have also revealed that older age, the severity of the disease tends to increase (Zhang et al., 2012). The probable reason is that immune function typically deteriorates with age, thereby accelerating the progression of the disease (Sun et al., 2014; Song et al., 2024). Regarding vasopressor therapy, the research by Tao et al. is in agreement with our findings. Their multicenter prospective study, employing a dual - model approach, discovered that the use of vasoconstrictor drugs is a risk factor for death in SFTS (Chen et al., 2025). The possible explanation is that SFTS patients with poor outcomes require vasoconstrictor drugs because of more severe symptoms such as fever and hypotension (Nie et al., 2020). The same situation applies to platelet transfusion. Finally, a high Ct value (low viral load) serves as a protective factor for SFTS, which aligns with the clinical trials and pathological reports of tocilizumab in the treatment of SFTS. In SFTS patients with a low viral load, the initial levels of cytokines like IL - 6 are also relatively low, and the inhibitory effect of tocilizumab is more pronounced (Yoo et al., 2022; Ge et al., 2024).

This study also has certain limitations. First, owing to the nature of a retrospective study, there may be a certain degree of selection bias, even though we used PSM to balance the baseline information of the tocilizumab therapy group and the control group. Second, since our research relies on the hospital’s case information system, it is not feasible to assess the direct effects of tocilizumab, such as the changes in cytokines and viral load after therapy. Third, this study is the absence of center-level and physician-level data, including hospital identifiers and detailed practice patterns. These unmeasured factors could influence both tocilizumab prescription and patient outcomes. Finally, AEs such as infections can be driven by severe disease itself or by concomitant treatments (e.g., antibiotics), and cannot be definitively attributed to tocilizumab alone.

## 5 Conclusion

In this multicenter retrospective cohort study, after PSM, tocilizumab use was associated with a higher recovery rate among SFTS patients. However, the difference between the groups in in-hospital mortality did not reach statistical significance after matching. For the selected AEs examined, including co-infection, the observed between-group differences did not reach statistical significance; these findings represent differences in retrospectively recorded events and should not be interpreted as evidence of safety. Multivariable logistic regression showed that age ≥ 65 years, vasopressor therapy, platelet transfusion, and Ct value were associated with prognosis among tocilizumab-treated patients, with vasopressor use and platelet transfusion likely reflecting underlying disease severity rather than independent prognostic determinants. Given the exploratory nature of these findings, prospective controlled studies are needed for confirmation.

## Data Availability

The original contributions presented in the study are included in the article/**Supplementary Material**. Further inquiries can be directed to the corresponding authors.

## References

[B1] Chen G. Chen T. Shu S. Ma K. Wang X. Wu D. . (2022). Expert consensus on diagnosis and treatment of severe fever with thrombocytopenia syndrome. Inf. Infect. Dis. 35, 385–393.

[B2] Chen Y. Wang H. Zhou F. Guo C. (2024). The use of glucocorticoid in severe fever with thrombocytopenia syndrome: a retrospective cohort study. Front. Cell. Infect. Microbiol. 14, 1419015. doi: 10.3389/fcimb.2024.1419015 39165922 PMC11333439

[B3] Chen T. Zhang M. Liu Q. Li W. Zeng Z. Chen C. . (2025). Development and validation of a novel mortality risk stratification simplified scoring scale for severe fever with thrombocytopenia syndrome. Clin. Microbiol. Infect. 31, 1378–1388. doi: 10.1016/j.cmi.2025.02.004 39922465

[B4] Cui N. Liu R. Lu Q. B. Wang L. Y. Qin S. L. Yang Z. D. . (2015). Severe fever with thrombocytopenia syndrome bunyavirus-related human encephalitis. J. Infect. 70, 52–59. doi: 10.1016/j.jinf.2014.08.001 25135231

[B5] Fajgenbaum D. C. June C. H. (2020). Cytokine storm. N. Engl. J. Med. 383, 2255–2273. doi: 10.1056/NEJMra2026131 33264547 PMC7727315

[B6] Ge H. H. Cui N. Yin X. H. Hu L. F. Wang Z. Y. Yuan Y. M. . (2024). Effect of tocilizumab plus corticosteroid on clinical outcome in patients hospitalized with severe fever with thrombocytopenia syndrome: a randomized clinical trial. J. Infect. 89, 106181. doi: 10.1016/j.jinf.2024.106181 38744376

[B7] Ge H. H. Wang G. Guo P. J. Zhao J. Zhang S. Xu Y. L. . (2022). Coinfections in hospitalized patients with severe fever with thrombocytopenia syndrome: a retrospective study. J. Med. Virol. 94, 5933–5942. doi: 10.1002/jmv.28093 36030552

[B8] Grupp S. A. Kalos M. Barrett D. Aplenc R. Porter D. L. Rheingold S. R. . (2013). Chimeric antigen receptor-modified T cells for acute lymphoid leukemia. N. Engl. J. Med. 368, 1509–1518. doi: 10.1056/NEJMoa1215134 23527958 PMC4058440

[B9] Hu L. F. Bian T. T. Chen Q. Liu M. Y. Li J. J. Kong Q. X. . (2024). Viral shedding pattern of severe fever with thrombocytopenia syndrome virus in severely ill patients: a prospective, multicenter cohort study. Heliyon 10, e33611. doi: 10.1016/j.heliyon.2024.e33611 39027598 PMC11255444

[B10] Hu Y. Y. Xia G. M. Zhang H. Zhang Z. H. (2025). Pathogen profile of co-infections and mortality determinants among patients with severe fever with thrombocytopenia syndrome. Front. Cell. Infect. Microbiol. 15, 1693567. doi: 10.3389/fcimb.2025.1693567 41446283 PMC12722868

[B11] Kang S. Y. Yoo J. R. Park Y. Kim S. H. Heo S. T. Park S. H. . (2023). Fatal outcome of severe fever with thrombocytopenia syndrome (Sfts) and severe and critical Covid-19 is associated with the hyperproduction of Il-10 and Il-6 and the low production of Tgf-B. J. Med. Virol. 95, e28894. doi: 10.1002/jmv.28894 37386895

[B12] Li H. Jiang X. M. Cui N. Yuan C. Zhang S. F. Lu Q. B. . (2021). Clinical effect and antiviral mechanism of T-705 in treating severe fever with thrombocytopenia syndrome. Signal. Transduct Target Ther. 6, 145. doi: 10.1038/s41392-021-00541-3 33859168 PMC8050330

[B13] Li H. Lu Q. B. Xing B. Zhang S. F. Liu K. Du J. . (2018). Epidemiological and clinical features of laboratory-diagnosed severe fever with thrombocytopenia syndrome in China, 2011-17: a prospective observational study. Lancet Infect. Dis. 18, 1127–1137. doi: 10.1016/s1473-3099(18)30293-7 30054190

[B14] Liu Y. Tong H. He F. Zhai Y. Wu C. Wang J. . (2023). Effect of intravenous immunoglobulin therapy on the prognosis of patients with severe fever with thrombocytopenia syndrome and neurological complications. Front. Immunol. 14, 1118039. doi: 10.3389/fimmu.2023.1118039 37033957 PMC10073413

[B15] Liu X. Zhang F. Qiao J. He W. (2025). Serum Il-6 and Il-10 levels are associated with fatal outcomes in patients with Sfts in China. J. Infect. Dev. Ctries 19, 273–279. doi: 10.3855/jidc.19939 40063754

[B16] Mihara M. Kasutani K. Okazaki M. Nakamura A. Kawai S. Sugimoto M. . (2005). Tocilizumab inhibits signal transduction mediated by both Mil-6r and Sil-6r, but not by the receptors of other members of Il-6 cytokine family. Int. Immunopharmacol. 5, 1731–1740. doi: 10.1016/j.intimp.2005.05.010 16102523

[B17] Nie Q. Wang D. Ning Z. Li T. Tian X. Bian P. . (2020). Analysis of severe fever with thrombocytopenia syndrome in critical ill patients in Central China. Shock 54, 451–457. doi: 10.1097/shk.0000000000001527 32097243

[B18] Rezaei Tolzali M. M. Noori M. Shokri P. Rahmani S. Khanzadeh S. Nejadghaderi S. A. . (2022). Efficacy of tocilizumab in the treatment of Covid-19: an umbrella review. Rev. Med. Virol. 32, e2388. doi: 10.1002/rmv.2388 36029180 PMC9539231

[B19] Scott L. J. (2017). Tocilizumab: a review in rheumatoid arthritis. Drugs 77, 1865–1879. doi: 10.1007/s40265-017-0829-7 29094311 PMC5736769

[B20] Seo J. W. Lee Y. M. Tamanna S. Bang M. S. Kim C. M. Kim D. Y. . (2024). Analysis of changes in viral load and inflammatory cytokines, as well as the occurrence of secondary infections, in Sfts patients treated with specific treatments: a prospective multicenter cohort study. Viruses 16, 1906. doi: 10.3390/v16121906 39772212 PMC11680452

[B21] Shankar-Hari M. Vale C. L. Godolphin P. J. Fisher D. Higgins J. P. T. Spiga F. . (2021). Association between administration of Il-6 antagonists and mortality among patients hospitalized for Covid-19: a meta-analysis. Jama 326, 499–518. doi: 10.1001/jama.2021.11330 34228774 PMC8261689

[B22] Song L. Zou W. Wang G. Qiu L. Sai L. (2024). Cytokines and lymphocyte subsets are associated with disease severity of severe fever with thrombocytopenia syndrome. Virol. J. 21, 126. doi: 10.1186/s12985-024-02403-0 38831352 PMC11149350

[B23] Sun H. Hu Q. Lu S. Yang Y. Zhang L. Long J. . (2025). Current status of severe fever with thrombocytopenia syndrome in China. Int. J. Mol. Med. 56, 169. doi: 10.3892/ijmm.2025.5610 40849814 PMC12373438

[B24] Sun L. Hu Y. Niyonsaba A. Tong Q. Lu L. Li H. . (2014). Detection and evaluation of immunofunction of patients with severe fever with thrombocytopenia syndrome. Clin. Exp. Med. 14, 389–395. doi: 10.1007/s10238-013-0259-0 24068614 PMC7101760

[B25] Sun Y. Jin C. Zhan F. Wang X. Liang M. Zhang Q. . (2012). Host cytokine storm is associated with disease severity of severe fever with thrombocytopenia syndrome. J. Infect. Dis. 206, 1085–1094. doi: 10.1093/infdis/jis452 22904342

[B26] Takahashi T. Maeda K. Suzuki T. Ishido A. Shigeoka T. Tominaga T. . (2014). The first identification and retrospective study of severe fever with thrombocytopenia syndrome in Japan. J. Infect. Dis. 209, 816–827. doi: 10.1093/infdis/jit603 24231186 PMC7107388

[B27] Yoo J. R. Kim T. J. Heo S. T. Hwang K. A. Oh H. Ha T. . (2021). Il-6 and Il-10 levels, rather than viral load and neutralizing antibody titers, determine the fate of patients with severe fever with thrombocytopenia syndrome virus infection in South Korea. Front. Immunol. 12, 711847. doi: 10.3389/fimmu.2021.711847 34484214 PMC8416084

[B28] Yoo J. R. Kim M. Kang M. J. Kim S. Lee K. H. Heo S. T. (2025). Tocilizumab for patients with severe fever with thrombocytopenia syndrome: Tocilizumab observational Sfts study-1. Yonsei Med. J. 66, 321–327. doi: 10.3349/ymj.2024.0209 40288904 PMC12041401

[B29] Yoo J. R. Lee K. H. Kim M. Oh H. J. Heo S. T. (2022). Tocilizumab therapy for Il-6 increment in a patient with non-fatal severe fever with thrombocytopenia syndrome. Int. J. Infect. Dis. 122, 656–658. doi: 10.1016/j.ijid.2022.06.058 35803471

[B30] Yu X. J. Liang M. F. Zhang S. Y. Liu Y. Li J. D. Sun Y. L. . (2011). Fever with thrombocytopenia associated with a novel bunyavirus in China. N. Engl. J. Med. 364, 1523–1532. doi: 10.1056/NEJMoa1010095 21410387 PMC3113718

[B31] Yun S. M. Lee W. G. Ryou J. Yang S. C. Park S. W. Roh J. Y. . (2014). Severe fever with thrombocytopenia syndrome virus in ticks collected from humans, South Korea, 2013. Emerg. Infect. Dis. 20, 1358–1361. doi: 10.3201/eid2008.131857 25061851 PMC4111194

[B32] Zhang S. S. Du J. Cui N. Yang X. Zhang L. Zhang W. X. . (2023). Clinical efficacy of immunoglobulin on the treatment of severe fever with thrombocytopenia syndrome: a retrospective cohort study. EBioMedicine 96, 104807. doi: 10.1016/j.ebiom.2023.104807 37738834 PMC10520313

[B33] Zhang Y. Z. He Y. W. Dai Y. A. Xiong Y. Zheng H. Zhou D. J. . (2012). Hemorrhagic fever caused by a novel bunyavirus in China: pathogenesis and correlates of fatal outcome. Clin. Infect. Dis. 54, 527–533. doi: 10.1093/cid/cir804 22144540

[B34] Zhang Y. Miao W. Xu Y. Huang Y. (2021). Severe fever with thrombocytopenia syndrome in Hefei: clinical features, risk factors, and ribavirin therapeutic efficacy. J. Med. Virol. 93, 3516–3523. doi: 10.1002/jmv.26544 32965706

[B35] Zuo Y. Wang H. Huang J. Zhang F. Lv D. Meng T. . (2023). Pulmonary infection in patients with severe fever with thrombocytopenia syndrome: a multicentre observational study. J. Med. Virol. 95, e28712. doi: 10.1002/jmv.28712 36991571

