# Supplementary material for: Clinical outcomes and safety of tocilizumab in severe fever with thrombocytopenia syndrome: a retrospective multicenter cohort study
Source: Front Cell Infect Microbiol. 2026 Sep 9;16:1894849. doi: 10.3389/fcimb.2026.1894849 (PMC13597313; doi:10.3389/fcimb.2026.1894849)
Supplement: Supplementary file 1 [file SupplementaryFile1.docx]

Supplementary materials

Figure S1 Comparison of data before and after propensity score match (PSM): (A): Histogram; (B): Jitter plot; (C): Love plot

**Table S1** the SMD of overall and baseline characteristics before and after PSM

| Variable | SMD-before PSM | SMD-after PSM |
| --- | --- | --- |
| Overall | 0.909 | 0.013 |
| Sex | -0.068 | -0.015 |
| Age | 0.041 | -0.018 |
| Viral load | -0.124 | 0.003 |
| Days from symptom onset to hospitalization | -0.088 | 0.015 |
| Comorbidities | 0.061 | -0.003 |
| Other diseases | 0.009 | 0.003 |
| Favipiravir therapy | 0.032 | 0.035 |
| Ribavirin therapy | -0.025 | -0.006 |
| Antibiotic therapy | 0.183 | -0.023 |
| Platelet transfusion | 0.247 | 0.047 |
| Clinical classification | 0.084 | -0.038 |

Note: standardized mean difference (SMD); Propensity Score Matching PSM.
